# Adjuvant TNF-α therapy to electrochemotherapy with intravenous cisplatin in murine sarcoma exerts synergistic antitumor effectiveness

**DOI:** 10.1515/raon-2015-0005

**Published:** 2015-03-03

**Authors:** Maja Cemazar, Vesna Todorovic, Janez Scancar, Ursa Lampreht, Monika Stimac, Urska Kamensek, Simona Kranjc, Andrej Coer, Gregor Sersa

**Affiliations:** 1 Department of Experimental Oncology, Institute of Oncology Ljubljana, Ljubljana, Slovenia; 2 Faculty of Health Sciences, University of Primorska, Izola, Slovenia; 3 Jozef Stefan Institute, Ljubljana, Slovenia

**Keywords:** TNF-α, electrochemotherapy, cisplatin, adjuvant immunotherapy

## Abstract

**Background:**

Electrochemotherapy is a tumour ablation modality, based on electroporation of the cell membrane, allowing non-permeant anticancer drugs to enter the cell, thus augmenting their cytotoxicity by orders of magnitude. In preclinical studies, bleomycin and cisplatin proved to be the most suitable for clinical use. Intravenous administration of cisplatin for electrochemotherapy is still not widely accepted in the clinics, presumably due to its lower antitumor effectiveness, but adjuvant therapy by immunomodulatory or vascular-targeting agents could provide a way for its potentiation. Hence, the aim of the present study was to explore the possibility of adjuvant tumour necrosis factor α (TNF-α) therapy to potentiate antitumor effectiveness of electrochemotherapy with intravenous cisplatin administration in murine sarcoma.

**Materials and methods:**

*In vivo* study was designed to evaluate the effect of TNF-α applied before or after the electrochemotherapy and to evaluate the effect of adjuvant TNF-α on electrochemotherapy with different cisplatin doses.

**Results:**

A synergistic interaction between TNF-α and electrochemotherapy was observed. Administration of TNF-α before the electrochemotherapy resulted in longer tumour growth delay and increased tumour curability, and was significantly more effective than TNF-α administration after the electrochemotherapy. Tumour analysis revealed increased platinum content in tumours, TNF-α induced blood vessel damage and increased tumour necrosis after combination of TNF-α and electrochemotherapy, indicating an anti-vascular action of TNF-α. In addition, immunomodulatory effect might have contributed to curability rate of the tumours.

**Conclusion:**

Adjuvant intratumoural TNF-α therapy synergistically contributes to electrochemotherapy with intravenous cisplatin administration. Due to its potentiation at all doses of cisplatin, the combined treatment is predicted to be effective also in tumours, where the drug concentration is suboptimal or in bigger tumours, where electrochemotherapy with intravenous cisplatin is not expected to be sufficiently effective.

## Introduction

Electrochemotherapy is a non-thermal tumour ablation modality that is safe and effective in any solid tumour type. Electrochemotherapy is based on the local delivery of electric pulses that permeabilize the cell membrane, allowing non-permeant or low-permeant anticancer drugs to enter the cell, thus augmenting their cytotoxicity by orders of magnitude. Treatment is safe, very well tolerated by the patients, and its efficacy is very high on the different tumour nodules.^1^ Electrochemotherapy has gained a role in routine clinical practice for treatment of cutaneous and subcutaneous tumours as local therapy.^2^,^3^ The development of specialized electrodes has made it possible to apply electrochemotherapy also to deep-seated tumours.^4^,^5^ The results of the first preclinical and clinical studies using specialized electrodes demonstrated feasibility of this approach for treatment of deep-seated tumours in liver and brain as well as for bone tumours and metastases.^4^,^6^–^11^

Among the drugs that have been tested in preclinical studies, bleomycin and cisplatin (CDDP) proved to be the most suitable for clinical use of electrochemotherapy. In the clinical use, both can be administered locally in the tumour, but only bleomycin systemically, intravenously, to obtain pronounced antitumor effect. Namely, in preclinical studies it was demonstrated that intratumoural administration of CDDP is more effective than systemic administration.^12^–^14^ The mechanisms of antitumor action of electrochemotherapy using both drugs are multifactorial, including anti-vascular effect and immune response stimulation.^15^,^16^ The activation of immune response is presumably due to the direct cytotoxic effect on tumour cells leading to immunological cell death.^16^ Hence, to potentiate antitumor effectiveness of systemic electrochemotherapy, we explored the possibility of additional intratumoural application of recombinant tumour necrosis factor α (TNF-α). TNF-α is a well-known pro-inflammatory cytokine with a wide range of biological functions.^17^ In addition to its immunomodulatory role, it also has an anti-vascular effect.^18^ To extend the application of electrochemotherapy with intravenous CDDP administration, addition of TNF-α could enhance the antitumour effectiveness of electrochemotherapy through its biological functions. Therefore, the aim of the present study was to explore the possibility of adjuvant intratumoural TNF-α therapy to electrochemotherapy with intravenous CDDP administration in murine sarcoma.

## Materials and methods

### Cell line

Murine fibrosarcoma cell line SA-1 (The Jackson Laboratory, Bar Harbor, ME, USA) was grown as monolayer in Eagle Minimum Essential Medium (EMEM) with Glutamax (Invitrogen, Paisley, UK), supplemented with 10% foetal calf serum (FCS) (Invitrogen) and gentamicin (30 μg/mL) (Invitrogen). Cells were routinely subcultured twice a week and incubated in an atmosphere with 5% CO_2_ at 37°C.

### Drug

CDDP (Medac, Wedel, Germany) was dissolved in sterile H_2_O. For each experiment, a fresh solution of CDDP was prepared. *In vitro*, the final concentrations of CDDP were prepared in EMEM. CDDP concentrations of 0.1 μg/mL and 0.5 μg/mL were used in the *in vitro* experiments.

Murine recombinant TNF-α was obtained from Affymetrix eBioscience (Santa Clara, CA, USA). The final concentrations of TNF-α were prepared in phosphate buffered saline (PBS) (Invitrogen). TNF-α concentrations 2.5×10^3^ U/mL, 2.5×10^4^ U/mL, and 2.5×10^5^ U/mL were used in the *in vitro* experiments.

### In vitro electroporation

Confluent cell cultures were trypsinized, washed in EMEM with FCS for trypsin inactivation and once in electroporation buffer (125 mM sacharose; 10 mM K_2_HPO_4_; 2.5 mM KH_2_PO_4_; 2 mM MgCl_2_·6H_2_O) at 4°C. The final cell suspension (22 × 10^6^ cells/mL) was prepared in electroporation buffer at 4°C. Cells (2 × 10^6^) were mixed with CDDP and/or TNF-α. Half of this mixture (1 × 10^6^ cells) was placed between two parallel stainless steel electrodes with a 2 mm gap in-between and subjected to eight square wave electric pulses (EP) with voltage to distance ratio 1300 V/cm, pulse duration 100 μs and frequency 1 Hz. EP were generated by generator of EP ELECTRO CELL B10 (Betatech, Saint-Orensde-Gameville, France). The other half of the mixture served as a control for CDDP and/or TNF-α treatment alone. After electroporation, cells were incubated at room temperature for 5 min, diluted in 2 mL of growth medium and then plated for the clonogenic assay.

### Clonogenic assay

Sensitivity of SA-1 cells exposed to CDDP or TNF-α alone, a combination of CDDP and TNF-α, EP alone, electrochemotherapy with CDDP (ECT), EP + TNF-α and ECT + TNF-α was determined by clonogenic assay. SA-1 cells were plated at a concentration of 200 cells/dish for exposure to CDDP or TNF-α alone and 500 cells/dish for combination of electroporation and CDDP or TNF-α. After 7 days colonies were fixed, stained with crystal violet and counted. The survival curve for the electrochemotherapy-treated cells was normalised for the cytotoxicity of EP treatment alone. The experiment was repeated twice in triplicates.

### Tumour and animal model

Murine fibrosarcoma SA-1 cells syngeneic to A/J mice (Harlan, Udine, Italy) were used in the study. Mice were maintained in a specific pathogen-free animal colony at constant room temperature (20–24°C), relative humidity (55±10%), and 12 hour light/dark cycle. Food and water were provided *ad libitum*. Animals were subjected to an adaptation period of 7–10 days before the experiments were carried out. All procedures on animals were performed in accordance with the official guidelines of European Union Directive 2010/63/EU and with the permission of the Ministry of Agriculture and the Environment of the Republic of Slovenia (permission no. 34401-4/2012/2) which was granted based on the approval of Ethical committee for animal experimentation of the Republic of Slovenia. At the beginning of the experiment, the animals were 9–11 weeks old. Solid tumours were induced by subcutaneous injections of SA-1 cells (5·10^5^ cells/0.1 mL 0.9% w/v NaCl) in the right flank prepared from the ascites from donor mouse. When the tumours reached approximately 40 mm^3^ in volume, the animals were randomly divided into experimental groups and subjected to a specific experimental protocol.

### *In vivo* electroporation

EP were delivered by generator of EP ELECTRO CELL B10 using flat parallel electrodes with 8 mm gap. Electrodes were placed percutaneously at the opposite tumour margins. Electroporation of tumours was performed by applying eight square-wave EP with voltage to distance ratio 1300 V/cm, pulse width 100 μs and repetition frequency 1 Hz with a perpendicular change of electrode orientation after 4 pulses. Good contact between the electrodes and the overlying skin was ensured by conductive gel (Parker Laboratories, Fairfield, NJ, USA).

### *In vivo* study design

**FIGURE 1.**
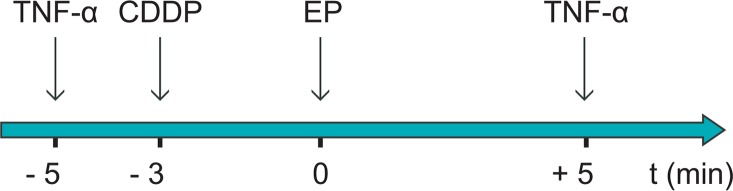
Time schedule of the treatment.

To evaluate the influence of TNF-α on the antitumour effectiveness of electrochemotherapy, TNF-α (1 × 10^5^ U) was applied intratumourally (i.t.) either 5 min before or after the application of EP. CDDP (4 mg/kg) was injected intravenously (i.v.) 3 min before the application of the electric pulses (Figure 1). The pertinent groups were control (untreated tumours), EP (application of EP only), CDDP (injection of CDDP only), TNF-α (injection of TNF-α only), ECT (electrochemotherapy with CDDP), EP + TNF-α −5 min (injection of TNF-α 5 min before the application of EP), EP + TNF-α +5 min (injection of TNF-α 5 min after the application of EP), CDDP + TNF-α −2 min (injection of TNF-α 2 min before the injection of CDDP), CDDP + TNF-α +8 min (injection of TNF-α 8 min after the injection of CDDP), ECT + TNF-α −5 min (injection of TNF-α 5 min before electrochemotherapy), and ECT + TNF-α +5 min (injection of TNF-α 5 min after electrochemotherapy).

In the second part of the *in vivo* study, the antitumour effect of TNF-α in combination with electrochemotherapy with different CDDP doses was evaluated. TNF-α was injected 2 min before the injection of CDDP (dose range 1 – 8 mg/kg) and 5 min before the application of electric pulses.

After the therapy, tumour growth was followed up by measurements of tumour diameters with a digital calliper. Tumour volume was calculated by the formula V=*a*·*b*·*c*·*π/6*, where a, b and c were three mutually orthogonal tumour diameters. From the calculated volume, arithmetic means and standard error of the means were calculated for each experimental group. Tumour doubling time was determined for each individual tumour, and tumour growth delay was calculated from the mean doubling time of experimental groups. When the tumour volume reached 300 mm^3^, animals were considered incurable and were humanely euthanized. When the tumours became impalpable, the response to treatment was scored as complete response. Mice that were in complete response 100 days after the treatment were considered as cured. In addition, the weight of the mice was followed as a general toxicity index.

### Determination of platinum content in tumours after treatment

Mice were sacrificed 24 h after treatment with CDDP (4 mg/kg), electrochemotherapy, combination of TNF-α and CDDP, and combination of TNF-α and electrochemotherapy. Tumours were excised and removed from the overlying skin. To determine platinum content in tumours after treatment, tumours were weighed, placed in 15-mL graduated polyethylene tubes and digested in 0.5 mL 65% nitric acid by incubation at 37°C for 2 days to obtain a clear solution. Platinum content in the samples was determined by electrothermal atomic absorption spectrometry on a Hitachi Z-8270 Polarized Zeeman Atomic Absorption Spectrometer, adjusted to a wavelength of 265.9 nm.

### Tumour histology

Mice were sacrificed 24 h after treatment with CDDP, electrochemotherapy, combination of TNF-α and CDDP, and combination of TNF-α and electrochemotherapy. Tumours were excised, removed from the overlying skin and fixed in IHC zinc fixative (BD Pharmingen, BD Biosciences, San Diego, CA, USA) for 24 h at room temperature, and embedded in paraffin. Two series of 2 μm thick tissue sections were cut from each paraffin block with step thickens of 20 μm.

First series of tissue sections were used for immunohistochemical staining of CD31, a marker for endothelial cells. Sections were incubated with rabbit polyclonal antibodies against murine CD31 (ab28364, Abcam, Cambridge, MA, USA) at dilution 1:1000. A peroxidase-conjugated streptavidin–biotin system (Rabbit specific HRP/DAB detection IHC kit, ab64261, Abcam) was used as the colorogenic reagent followed by haematoxylin counterstaining. The immunohistochemically stained sections were examined in blind fashion under light microscopy and 5 images of viable tumour tissue from the same tumour section were captured with a DP72 CCD camera (Olympus, Hamburg, Germany) connected to a BX-51 microscope (Olympus).

Second series of tissue sections were stained with haematoxylin and eosin for evaluation of tumour necrosis. These slides were examined under the microscope Olympus BX-51 and visual light images were recorded with a digital camera DP72 CCD (Olympus). Digital images of haematoxylin and eosin stained tumour sections were analysed in blind fashion and percentage of necrosis was estimated by 4 independent observers.

### Statistical analysis

SigmaPlot 11.0 (Systat Software Inc., San Jose, CA, USA) was used for statistical analysis. Normal distribution of the data was tested using Shapiro-Wilk test. All pairwise multiple comparisons for normally distributed data were tested with Holm-Sidak test after One Way ANOVA. All pairwise multiple comparisons for data that was not normally distributed were tested using Tukey test or Dunn’s test (if treatment group sizes were unequal) after Kruskal-Wallis One-Way ANOVA on ranks. Differences were considered significant when p value was less than 0.05.

## Results

### TNF-α does not potentiate CDDP cytotoxicity

**FIGURE 2.**
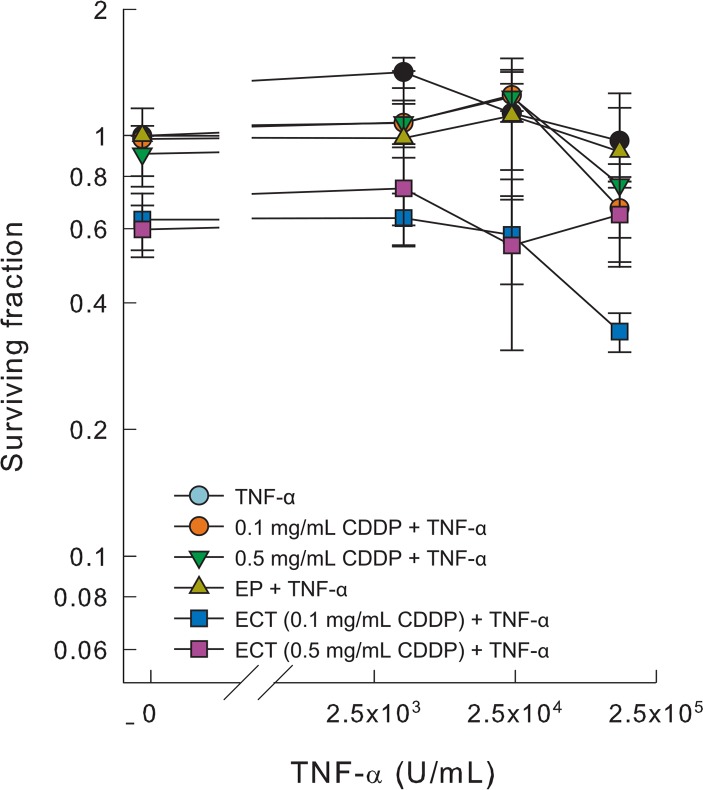
Cell survival after electrochemotherapy with TNF-α at different doses. The surviving fraction of cells exposed to electrochemotherapy was normalized to electric pulses alone. Symbols represent median ± MAD.

*In vitro* sensitivity of SA-1 cells to electrochemotherapy with or without TNF-α was determined by clonogenic assay (Figure 2). TNF-α in all the selected concentrations up to 2.5·10^5^ U/mL did not exert any direct cytotoxic effect on SA-1 cells *in vitro*. Furthermore, no interaction of TNF-α with CDDP or electrochemotherapy was noticed at both concentrations of CDDP tested.

### Adjuvant TNF-α before or after ECT *in vivo*?

To determine if TNF-α can potentiate antitumor effectiveness of electrochemotherapy with intravenous CDDP injection *in vivo*, we evaluated the effect of intratumoural TNF-α injection before or after electrochemotherapy on tumour growth (Table 1). A relatively low CDDP dose of 4 mg/kg was selected in order to be able to demonstrate the possible interaction with adjuvant TNF-α therapy.

Among all the combined modality groups only the adjuvant TNF-α therapy to electrochemotherapy produced significant tumour growth delay compared to control tumours and resulted in tumour cures. In these combined treatment groups, tumour doubling times were greater than the sum of tumour doubling times of single therapies (Table 1). Specifically, the treatment of tumours before the electrochemotherapy was significantly more effective (p<0.05) than treatment with TNF-α after electrochemotherapy, producing longer tumour growth delay, and higher tumour curability rate (36% vs. 27%, respectively) (Table 1). The single or combined therapies did not have toxic side effects or did not induce animal weight loss for more than 10% of the body weight (data not shown).

### Platinum content in tumours after treatment

**FIGURE 3.**
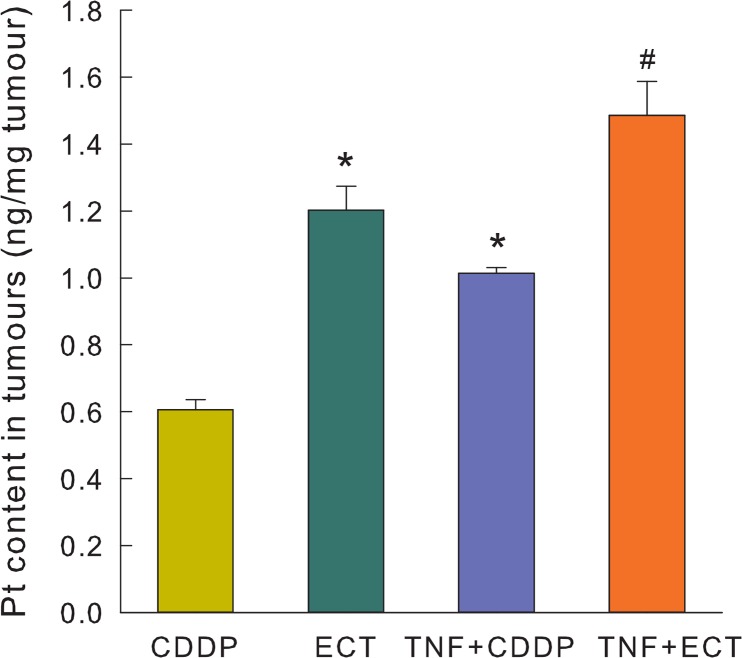
Platinum content in tumours 24 h after different treatments. Increased platinum uptake was observed after electrochemotherapy and exposure to TNF-α. Bars represent mean ± SEM. * p<0.05 vs. CDDP, # p<0.05 vs. CDDP, ECT and TNF+CDDP.

To determine whether TNF-α treatment affects CDDP uptake in tumours, platinum content in whole tumours was determined 24 h post treatment. Significant differences in platinum concentration in tumours were observed after different treatments (Figure 3). Tumour treatment with electrochemotherapy alone or with TNF-α resulted in significantly higher platinum concentration in the whole tumours (p<0.05). Electrochemotherapy increased platinum content in the tumours, as expected, approximately by factor of 2.^19^ Furthermore, in the TNF-α with the electrochemotherapy treatment group, platinum content in tumours was further significantly increased compared to tumours treated with either electrochemotherapy or TNF-α in combination with CDDP without EP. Overall 2.2-fold increase in tumour platinum content was observed. The data do not prove the internalisation of the platinum in the cells of tumours, but rather indicate on the overall platinum content in the tumours.

### Histological analysis

**FIGURE 4.**
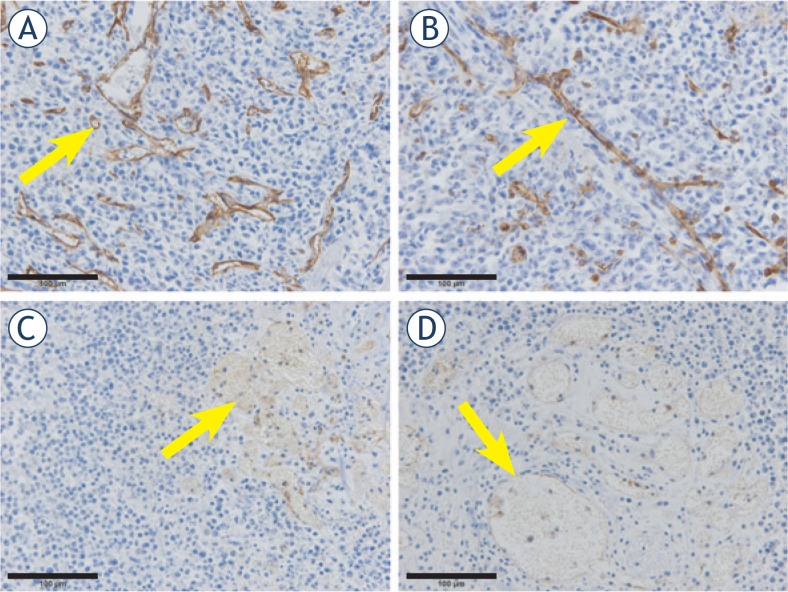
Vascular effect of different treatments. Anti-CD31 staining of tumour sections in tumours 24 h after different treatments at 40× magnification. **(A)** A typical tumour blood vessels network with thin vessels walls consisting only of endothelial cells was observed after CDDP alone (arrow). **(B)** After electrochemotherapy vasoconstriction of blood vessels was observed (arrow). **(C)** Dilated tumour vessels with stacked erythrocytes were observed after combination of TNF-α and CDDP (arrow). **(D)** After combination of TNF-α and electrochemotherapy only few large dilated blood vessels with mostly hyperaemic and occasionally damaged blood vessels were observed. **(A)** – CDDP; **(B)** – electrochemotherapy; **(C)** – TNF-α and CDDP; **(D)** – TNF-α and electrochemotherapy). Scale bar 100 μm.

The effect on tumour blood vessels was demonstrated by immunohistochemical analysis of anti-CD31-stained blood vessels in viable regions of tumours 24 h after treatments (Figure 4). Differences were observed in tumours treated with either CDDP or electrochemotherapy in comparison to tumours treated with TNF-α in combination with CDDP or electrochemotherapy. A strong anti-CD31 staining was observed in tumours treated with either CDDP or electrochemotherapy, while anti-CD31 staining in tumours treated with TNF-α was much weaker. In tumours treated with CDDP alone, typical tumour blood vessels network with thin vessels walls consisting only of endothelial cells was observed (Figure 4A). After electrochemotherapy, most of the blood vessels were narrowed indicating clearly seen vasoconstriction (Figure 4B). Number of functional blood vessels was significantly reduced in tumours after treatment with TNF-α. In tumours treated with combination of TNF-α and CDDP, tumour vessels were dilated, with stacked erythrocytes (Figure 4C). This effect was more pronounced after treatment with combination of TNF-α and electrochemotherapy, where only few large dilated blood vessels with mostly hyperaemic and occasionally damaged blood vessels and in some areas also extravasated erythrocytes were observed (Figure 4D).

**FIGURE 5.**
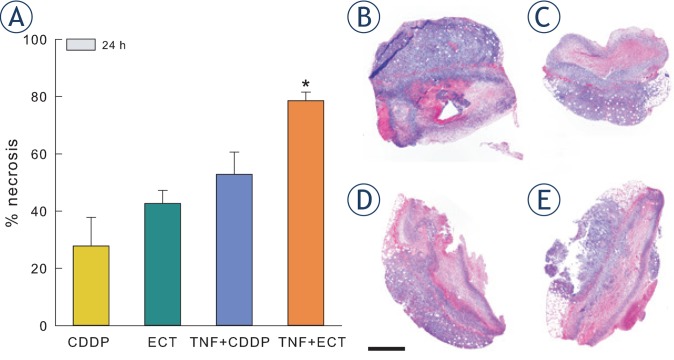
Percentage of necrosis in tumours 24 h after different treatments. **(A)** Increased percentage of necrotic tumour area was observed 24 h after treatment with combination of TNF-α and electrochemotherapy. (B–E) Haematoxylin and eosin staining of tumour sections in tumours 24 h after different treatments at 4× magnification. **(B)** – CDDP; **(C)** – electrochemotherapy; **(D)** – TNF-α and CDDP; **(E)** – TNF-α and electrochemotherapy. Scale bar 1 mm. Bars represent mean ± SEM. * p<0.05 vs. CDDP, ECT, and TNF + CDDP.

Necrosis of tumour tissue was evaluated histologically in all samples 24 h after different treatments (Figure 5). Cisplatin treatment did not result in significantly increased necrosis compared with control untreated tumours.^19^ The degree of necrosis was significantly increased in tumours treated with adjuvant TNF-α and electrochemotherapy in comparison to tumours treated with CDDP, electrochemotherapy or combination of TNF-α and CDDP.

### Is TNF-α interaction with electrochemotherapy CDDP dose dependent?

In the second part of the study, the effect of combined TNF-α and electrochemotherapy with different CDDP doses (1–8 mg/kg) on tumour growth was evaluated. Based on the previous experiment, adjuvant TNF-α therapy was given before electrochemotherapy.

**FIGURE 6.**
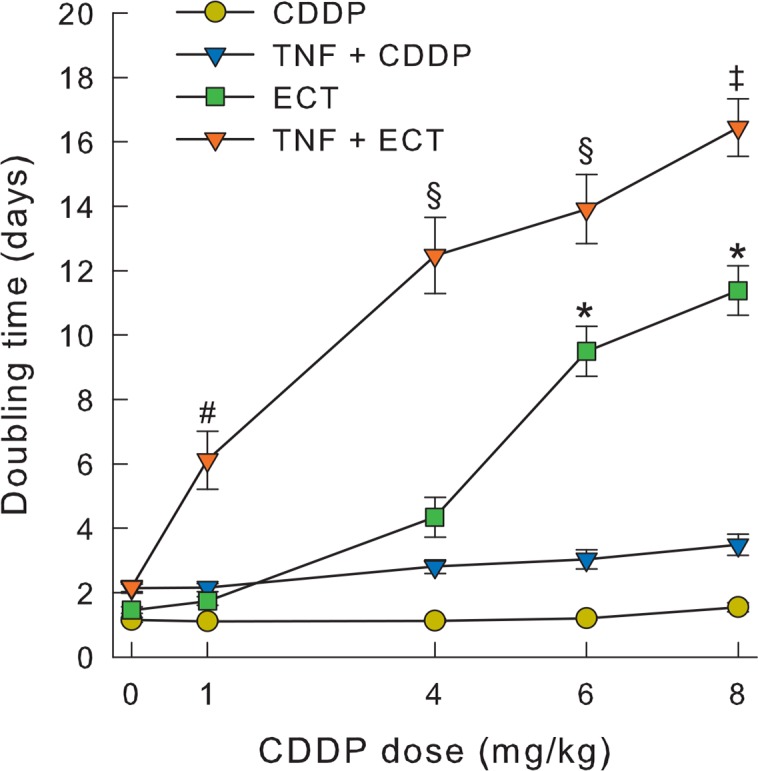
*In vivo* application of TNF-α and electrochemotherapy with different CDDP doses. Tumour doubling times after treatment with TNF-α and electrochemotherapy with different doses of CDDP were increased in comparison to electrochemotherapy alone. Symbols represent mean ± SEM. * p<0.05 vs. EP, ECT 1 mg/kg and ECT 4 mg/kg; # p<0.05 vs. TNF-α + EP; § p<0.05 vs. TNF-α + EP and TNF-α + ECT 1 mg/kg; ‡ p<0.05 vs. TNF-α + EP and TNF-α + ECT 1–4 mg/kg.

As predicted, electroporation significantly potentiated CDDP effectiveness, which was CDDP dose dependent (Figure 6). Electrochemotherapy with the CDDP doses of 6 and 8 mg/kg produced significant tumour growth delay of 8.4 ± 0.8 and 10.3 ± 0.8 days. The adjuvant intratumoural TNF-α therapy significantly potentiated the effectiveness of electrochemotherapy at all the CDDP doses used, also at the lowest CDDP dose of 1 mg/kg that had no effect in electrochemotherapy. Synergistic effect of combined treatment modality was observed at lower CDDP doses (1 and 4 mg/kg), where tumour doubling time of combined treatment modality was greater than the additive effect of single therapies. Addition of TNF-α to electrochemotherapy prolonged tumour doubling time for 5.5 days in comparison to electrochemotherapy treatment alone throughout the tested concentration range. This synergistic effect of the therapies is supported by the fact that only the tumours in combined modality protocol were cured and tumours treated with electrochemotherapy alone were not. Namely, after combined treatment of TNF-α and electrochemotherapy with 6 mg/kg CDDP, 2 mice (9.5%) were cured, and after combined treatment of TNF-α and electrochemotherapy with 8 mg/kg CDDP, 1 mouse (4.3%) was cured.

## Discussion

In the study, the antitumour effectiveness of TNF-α combined with electrochemotherapy with intravenous CDDP was evaluated in murine fibrosarcoma tumour model. Adjuvant immunotherapy with TNF-α preceding electrochemotherapy, increased antitumor effectiveness of electrochemotherapy with intravenous CDDP. Prolonged tumour growth and increased tumour curability were observed after combined treatment with TNF-α and electrochemotherapy with intravenous CDDP. The underlying mechanism is most likely vascular targeted effect of TNF-α combined with its immunomodulatory effect, as previously proposed for combination with electrochemotherapy with bleomycin.^16^

Electrochemotherapy gained clinical recognition in the treatment of cutaneous tumours or metastases as well as deep-seated tumours.^2^–^4^,^6^–^11^ Currently, bleomycin is the drug of choice for majority of the electrochemotherapy treatments, although CDDP is also in Standard Operating Procedures, but only if applied intratumoural. CDDP applied intravenously has not been recognized to be sufficiently effective.^1^ However, this was based on a report where EP were applied on large tumours that were not expected to respond to systemic chemotherapy in metastatic melanoma patients.^13^ Due to the size of these treated tumours (the longest diameter of tumours mainly exceeded 3 cm) the response was not comparable to bleomycin data, where predominantly tumours less than 3 cm in diameter were treated.^20^–^23^ Nevertheless, it is expected that with time CDDP will gain recognition, predominantly in such situations as in the first study^13^, or when its effectiveness could be potentiated with adjuvant treatment.

Recently, it was demonstrated that tumours larger than 3 cm in size have lower response rate after single electrochemotherapy treatment than smaller ones.^22^,^24^,^25^ Furthermore, in preclinical studies, it was shown that electrochemotherapy with bleomycin induces immunological cell death^16^, providing additional rational for combination with immunotherapy. Therefore, one way to potentiate electrochemotherapy effectiveness is adjuvant immunotherapy using different cytokines, such as interleukin 2 and 12, granulocyte-monocyte colony stimulating factor, and TNF-α.^26^–^30^ All these studies have provided evidence that electrochemotherapy with bleomycin can be potentiated with adjuvant immunotherapy, demonstrating increased tumour growth delay as well as tumour curability. It was already demonstrated that electrochemotherapy with bleomycin can be effectively potentiated in sarcoma model by TNF-α^30^, however, combination of electrochemotherapy with CDDP with adjuvant immunotherapy has not been tested yet.

TNF-α is a well-known pro-inflammatory cytokine with a wide range of biological functions, including an immunomodulatory role and antivascular action.^17^,^18^ Due to its side effects, Its intravenous administration is not recommended^31^, therefore we decided to use intratumoural route and combined it with electrochemotherapy with intravenous CDDP administration.

Our results demonstrated potentiation of antitumour effectiveness of electrochemotherapy in combination with TNF-α *in vivo*. A synergistic interaction between TNF-α and electrochemotherapy with intravenous CDDP administration was observed. This synergistic interaction cannot be attributed to TNF-α cytotoxicity to tumour cells due to the lack of the effect *in vitro*. Contrary to early speculations of TNF-α cytotoxic action on malignant cells in animal tumour models, it is now accepted that TNF-α is weakly cytotoxic to malignant cells.^32^ Our *in vitro* results support this, as no cytotoxic effect of TNF-α alone or in combination with electric pulses on SA-1 fibrosarcoma cells was observed. Similarly, no cytotoxic effect of TNF-α in combination with EP for electrochemotherapy was observed in our previous study.^30^ Therefore, it is more likely that cell types other than tumour cells, such as endothelial cells or immune cells found in the tumour stroma are responsible for the observed antitumour effect.

*In vivo*, two distinct and successive effects of TNF-α on tumour-associated vasculature are known. Firstly, TNF-α increases vascular endothelium permeability, resulting in reduction of interstitial fluid pressure and improved penetration of chemotherapeutic agents within the tumour tissue, and secondly, TNF-α causes tumour vessel destruction.^33^–^36^ The mechanism whereby these events occur involves rapid perturbation of cell-cell adhesive junctions, leading to loss of intercellular adhesion, and inhibition of αvβ3 integrin signalling in tumour-associated vessels.^36^ Specifically, it selectively damages the integrity of tumour vasculature by disrupting VE-cadherin complexes and thus creates gaps between endothelial cells.^37^ In addition, the expression of TNF-α receptors is speculated to be up-regulated in tumour vessels in comparison to normal vessels, which makes tumour blood vessels specific targets for TNF-α treatment.^38^ Binding of TNF-α to its receptors on endothelial cells triggers apoptosis and leads to hyperpermeability of the tumour blood vessels, and extravasation of blood cells.^38^ In our study, anti-vascular action of TNF-α was confirmed by histological analysis of tumour sections. TNF-α treatment damaged existing blood vessels, resulting in weak anti-CD31 staining. In addition, number of functional blood vessels was significantly reduced after exposure to TNF-α. Blood vessels were dilated with stacked erythrocytes. This effect was even more pronounced after TNF-α combined with electrochemotherapy, where also extravasated erythrocytes were observed. In addition, overall platinum content in tumours was increased 24 h after treatment with TNF-α compared to tumours treated with CDDP alone indicating facilitated uptake of CDDP in TNF-α treated tumours. This correlates well with other studies where improved penetration and increased uptake of anticancer drugs was observed in combination with TNF-α.^39^,^40^

Taken together, our results indicate indirect antitumour action of adjuvant TNF-α in SA-1 fibrosarcoma tumours treated with ECT with low CDDP doses. This antitumour action of TNF-α is likely due to hyperpermeability of tumour blood vessels, leading also to increased accumulation of CDDP in the tumours. Based on these results, we speculate that the mechanism of action of adjuvant TNF-α in electrochemotherapy treated tumours is primarily through a vascular action. Nonetheless, especially at higher CDDP doses, at which tumour cures were obtained, involvement of activated immune response should be taken into account. Namely, in clinical settings, induction of inflammatory response after TNF-α based isolated limb perfusion was observed in the tumour tissue.^36^ However, involvement of non-immunological antitumour mechanism of TNF-α is also supported by our previous study, where we showed antitumour effect of TNF-α also in immunosuppressed mice.^41^ Indeed, in our study, we did not detect the significant infiltration of immune cells into the tumours in histological section, most probably due to the early time of tumours excision after the therapy. Namely, the vascular effect of TNF-α is rapid, while immune adaptive response of the organisms needs the time to develop. Nevertheless, tumour curability after the combined treatment should be attributed to the involvement of adaptive immune system. We speculate that by replacing the recombinant TNF-α with local TNF-α gene therapy, which would result in longer persistence of therapeutic TNF-α concentrations in organism, the immunological components of the synergistic effect would account for higher curability rate.

In conclusion, adjuvant intratumoural TNF-α therapy synergistically contributes to electrochemotherapy with intravenous CDDP administration. Due to its potentiation at all doses of CDDP, the combined treatment is predicted to be effective also in less perfused areas of tumours, where the drug accumulation is not optimal, or in bigger tumours, where the effectiveness of electrochemotherapy with intravenous CDDP is not expected to be sufficiently effective. Its synergistic effectiveness can be attributed to actions of TNF-α; vascular and also immunomodulatory, that could be further potentiated by replacing the injection of recombinant TNF-α protein with application of gene therapy with TNF-α.

## Figures and Tables

**TABLE 1.** Tumour doubling times and growth delay after different treatments. TNF-α was applied either before (TNF-α +) or after (TNF-α −) treatment

**Treatment group**	**n**	**Doubling time (mean ± SEM)**	**Growth delay (vs. control)**	**Cures**
Control	7	2.2 ± 0.1		
EP	7	3.2 ± 0.4	1.0	
CDDP	7	3.1 ± 0.2	0.9	
TNF-α	7	2.9 ± 0.1	0.7	
ECT	8	4.1 ± 0.9	1.9	
TNF-α −5 min + EP	10	7.2 ± 0.4	5.0	
TNF-α +5 min + EP	10	5.8 ± 0.5	3.6	
TNF-α −2 min + CDDP	10	3.7 ± 0.4	1.5	
TNF-α +8 min + CDDP	10	3.5 ± 0.3	1.3	
TNF-α −5 min + ECT	11	23.7 ± 2.3^*‡^	21.5	4 (36%)
TNF-α +5 min + ECT	11	15.4 ± 1.5^*^	13.2	3 (27%)

* = Significantly increased doubling time in comparison to other treatment groups;

‡ = Significantly increased doubling time in comparison to TNF- α +5 min + ECT. p<0.05
